# Complete Genome Sequence of Spiroplasma monobiae MQ-1^T^ (ATCC 33825), a Bacterium Isolated from the Vespid Wasp (Monobia quadridens)

**DOI:** 10.1128/genomeA.00347-18

**Published:** 2018-05-03

**Authors:** Yi-Ming Tsai, Wen-Sui Lo, Pei-Shan Wu, Shu-Ting Cho, Chih-Horng Kuo

**Affiliations:** a Institute of Plant and Microbial Biology, Academia Sinica, Taipei, Taiwan

## Abstract

Spiroplasma monobiae MQ-1^T^ (ATCC 33825) was isolated from the hemolymph of an adult vespid wasp (Monobia quadridens) collected in Maryland. Here, we report the complete genome sequence of this bacterium to facilitate the investigation of its biology and the comparative genomics among Spiroplasma species.

## GENOME ANNOUNCEMENT

Spiroplasma monobiae is known to be associated with the vespid wasps (Hymenoptera: Vespidae) in North America (1). The type strain MQ-1^T^ was isolated from the hemolymph of an adult Monobia quadridens collected in Maryland and is the representative of group VII within the genus (2). Several interesting features were identified in the early characterization of this bacterium, including its possession of the smallest genome within the Apis clade (3, 4), its unique pattern of DNA methylation (5), and the potent effect of inducing tumor necrosis factor alpha secretion in mammalian cells by its membrane (6). To facilitate future investigation of the biology of this bacterium, as well as to improve the taxon sampling of available Spiroplasma sequences for comparative genomics and evolutionary studies (7), we determined its complete genome sequence.

The strain was acquired from the American Type Culture Collection (catalog number ATCC 33825). The freeze-dried sample was processed according to the manufacturer’s instructions and cultured in M1D medium (8) prior to DNA extraction using the Wizard genomic DNA purification kit (Promega, USA). PCR and Sanger sequencing were performed to verify that the 16S rRNA gene sequence matched the reference record (GenBank accession number GU585673).

The procedures for genome sequencing, assembly, and annotation were based on those described in our previous studies (9–20). Briefly, the Illumina MiSeq platform was used to generate raw reads from one paired-end library (∼255-bp insert; ∼600-fold coverage). The initial *de novo* assembly was performed using Velvet version 1.2.10 (21). Subsequently, PAGIT version 1 (22) was used to assist an iterative process for improving the assembly. For each iteration, the raw reads were mapped to the assembly using the Burrows-Wheeler transform version 0.7.12 (23), programmatically checked using the MPILEUP program in SAMtools package version 1.2 (24), and visually inspected using Integrative Genomics Viewer (IGV) version 2.3.57 (25). Polymorphic sites and gaps were corrected based on the mapped reads. The process was repeated until the complete genome sequence was obtained. The programs RNAmmer (26), tRNAscan-SE (27), and Prodigal (28) were used for gene prediction. The gene names and product descriptions were first annotated based on the homologous genes in other Spiroplasma genomes (9–20) as identified by OrthoMCL (29). Subsequent manual curation was based on the information obtained from the BlastKOALA tool (30) and BLASTp (31) searches against the NCBI nonredundant database (32). Putative clustered regularly interspaced short palindromic repeats (CRISPRs) were identified using CRISPRFinder (33).

The complete genome sequence of Spiroplasma monobiae MQ-1^T^ consists of one circular chromosome that is 891,575 bp in size with a G+C content of 27.8%; no plasmid was found. The first version of the annotation includes one set of 16S-23S-5S rRNA genes, 29 tRNAs (covering all 20 amino acids), 813 protein-coding genes, and 2 pseudogenes; no CRISPR locus was found.

### Accession number(s).

The complete genome sequence of Spiroplasma monobiae MQ-1^T^ has been deposited at DDBJ/EMBL/GenBank under the accession number CP025543.
